# Investigation of Electron Transport Layer Influence on Asymmetric Bipolar Switching in Transparent BST-Based RRAM Devices

**DOI:** 10.3390/mi16111302

**Published:** 2025-11-20

**Authors:** Kai-Huang Chen, Ming-Cheng Kao, Hsin-Chin Chen, Yao-Chin Wang, Chien-Min Cheng, Wei-Min Xu

**Affiliations:** 1 Department of Electronic Engineering, Cheng Shiu University, Kaohsiung 83347, Taiwan; 0662@gcloud.csu.edu.tw (H.-C.C.); 0644@gcloud.csu.edu.tw (Y.-C.W.); 2 Graduate Institute of Aeronautics, Department of Information and Communication Engineering, Chaoyang University of Technology, Taichung 413310, Taiwan; 3 Department of Electronic Engineering, Southern Taiwan University of Science and Technology, Tainan 71005, Taiwan; ccmin523@gmail.com (C.-M.C.); ma930104@stust.edu.tw (W.-M.X.)

**Keywords:** bipolar switching characteristics, electron transport layer, BST, resistance random access memory, electronic conduction mechanism, *I–V*

## Abstract

Ba_0.6_Sr_0.4_TiO_3_ (BST) thin films were deposited on ITO substrates via rf magnetron sputtering, followed by structural and morphological characterization using XRD and FE-SEM. Metal–insulator–metal (MIM) RRAM devices were fabricated by depositing Al top electrodes, and their electrical properties were examined through *I–V* measurements. The optimized BST films deposited at 40% oxygen concentration exhibited stable resistive switching, with an operating voltage of 3 V, an on/off ratio of 1, and a leakage current of 10^−8^ A. After rapid thermal annealing at 500 °C, the on/off ratio improved to 2 but leakage increased to 10^−3^ A. Incorporating an electron transport layer (ETL) effectively suppressed the leakage current to 10^−5^ A while maintaining the on/off ratio at 2. Moreover, a transition from bipolar to unipolar switching was observed at higher oxygen concentration (60%). These results highlight the role of ETLs in reducing leakage and stabilizing switching characteristics, providing guidance for the development of transparent, low-power, and high-reliability BST-based RRAM devices. This study aims to investigate the role of Ba_0.6_Sr_0.4_TiO_3_ (BST) ferroelectric oxide as a functional switching layer in resistive random-access memory (RRAM) and to evaluate how interface engineering using an electron transport layer (ETL) can improve resistive switching stability, leakage suppression, and device reliability.

## 1. Introduction

With the rapid advancement of modern technology, electronic products have become indispensable in daily life, continuously evolving with new generations of devices and 3C applications to meet user demands. Consumers increasingly expect electronic devices to offer multifunctionality, fast response times, long operational lifetimes, lightweight portability, and compact designs. Among these demands, memory performance—particularly in terms of access speed, storage capacity, and functional applications—has emerged as a critical core technology. To date, numerous types of memory have been developed, which can be broadly categorized into volatile memory and non-volatile memory. Volatile memories require a constant power supply to retain data, and the stored information is lost once power is removed. Representative examples include dynamic random-access memory (DRAM) and static random-access memory (SRAM). These devices, with their simple structures, fast access speeds, and low power consumption, are well-suited for assisting processors in high-speed computing tasks [1,2]. In contrast, non-volatile memories are capable of retaining stored data even in the absence of power, allowing information to be retrieved once power is reapplied. Various next-generation non-volatile memory technologies have been developed, including magnetoresistive random-access memory (MRAM), phase-change random-access memory (PCRAM), ferroelectric random-access memory (FeRAM), and resistive random-access memory (RRAM). Among these, RRAM has attracted the most attention due to its promising characteristics, such as fast programming/erasing speed, simple metal–insulator–metal (MIM) structure, low operating voltage, and low power consumption. These unique advantages have motivated extensive research efforts, with many scholars actively exploring RRAM mechanisms and applications for integration into future high-performance electronic devices [3,4,5,6,7,8,9,10,11,12].

Resistive random-access memory (RRAM) is a novel type of non-volatile memory that operates by modulating the resistance of the insulating layer through an applied voltage. Its structure is based on a simple metal–insulator–metal (MIM) configuration, which is highly scalable and can be readily integrated with either a diode to form a 1D1R structure or a transistor to form a 1T1R structure. This memory technology enables ultrafast switching (in the nanosecond range) between the high-resistance state (HRS) and the low-resistance state (LRS), with the binary states “0” and “1” being represented by the HRS and LRS, respectively. Furthermore, RRAM can be operated under low voltage and current levels (in the range of μA to nA), while exhibiting stable endurance (cycling performance) and excellent retention characteristics [13,14,15,16,17,18,19]. In past studies, the intrinsic *I–V* and C–V characteristics of ABO_3_ ferroelectric oxides were extensively investigated in the context of non-volatile ferroelectric RAM devices. More recently, alternative ABO_3_-based oxides such as BaSrTiO_3_ (BST) and SrBiTiO_3_ (SBT) thin films have attracted increasing attention for application in resistive random-access memory (RRAM) devices, owing to their exceptionally high resistive switching ratios and wide memory windows, which are highly desirable for future Internet of Things (IoT) applications. According to the recent literature, the controlled incorporation of Mn dopants has been shown to effectively enhance switching speed, expand the memory window, increase endurance, and reduce electron density within the memory layer, thereby improving overall device performance [20,21,22,23,24].

In this work, perovskite-based materials are employed as the insulating layer in RRAM devices, with the addition of an electron transport layer (ETL) to investigate the underlying physical mechanisms. Titanium dioxide (TiO_2_) was selected as the ETL material, aiming to achieve two functions. For an applied bias, TiO_2_ can undergo partial metallization, forming a metallic-like state that facilitates conduction, and it serves as an electron-blocking layer, effectively accumulating carriers at the interface. Electrical characterization and conduction mechanism analysis were performed to elucidate these effects. The specific research objectives were focused as follows: investigating the influence of oxygen concentration on the crystal structure and thin-film quality, examining the effect of annealing temperature on the electrical properties, and analyzing the changes in device characteristics induced by the introduction of the electron transport layer. Although many RRAM systems based on metal oxides (e.g., HfO_2_, TiO_2_) have been reported, these materials often suffer from unstable filament formation, limited endurance, and large device-to-device variation, particularly under high-field operation. BST, a perovskite ferroelectric oxide, offers high dielectric permittivity, polarization-assisted charge transport, and robust thermal stability, which can effectively address these bottlenecks. Furthermore, incorporating a TiO_2_-based ETL can modulate interfacial barrier height and enhance filament controllability, leading to improved switching uniformity and reduced power consumption. This study aims to develop a BST-based RRAM structure with improved reliability through interface modification—providing insight into material selection and device optimization for next-generation non-volatile memory technologies.

## 2. Experiment Detail

In this study, Ba_0.6_Sr_0.4_TiO_3_ (BST) thin films were deposited as the insulating layer in metal–insulator–metal (MIM) resistive random-access memory (RRAM) devices using radio-frequency (rf) magnetron sputtering. A 3-inch Ba_0.6_Sr_0.4_TiO_3_ (BST) ceramic target was positioned at a target-to-substrate distance of approximately 5 cm to ensure uniform film deposition. Prior to deposition, a 20 min pre-sputtering process was carried out in a pure argon (Ar) atmosphere to remove intrinsic defects from the target surface and stabilize the plasma. The deposition was performed at room temperature with the following parameters: rf power of 100 W, chamber pressure of 20 mTorr, and sputtering time of 10 min, under varying oxygen-to-argon gas ratios. The Ba_0.6_Sr_0.4_TiO_3_ (BST) films were deposited onto ITO/glass substrates to form the insulating layer. Post-deposition, the films were subjected to rapid thermal annealing (RTA) at 500 °C for 30 s to improve crystallinity and relieve residual stress. Here, the specified 30 s corresponds to the dwell time at the target temperature, excluding heating and cooling. The heating rate was 20 °C/s, followed by a 30 s hold, and natural cooling at 15 °C/s until the temperature decreased below 100 °C, after which samples were removed. Additionally, for Ba_0.6_Sr_0.4_TiO_3_ (BST) films RRAM devices within the electron transport layer (ETL) process, the deposition was performed at room temperature with the following parameters: rf power of 100 W, chamber pressure of 3 mTorr, sputtering time of 10 min, and 40% oxygen-to-argon gas ratios. For the electron transport layer (ETL) process, the deposition parameters of rf power of 100 W, chamber pressure of 3 mTorr, sputtering time of 5 min, and 40% oxygen-to-argon gas ratios were prepared.

**Figure 1:**
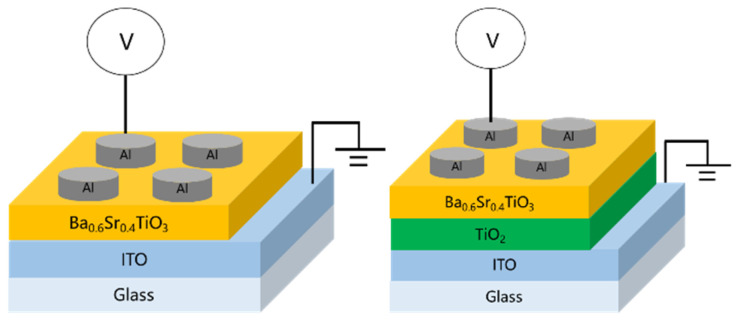
Al/BST/ITO/glass and Al/BST/TiO_2_/ITO/glass structure RRAM device diagram.

Subsequently, circular top electrodes (diameter: 0.1 cm) of aluminum (Al) were deposited using a shadow mask and dc sputtering in a pure argon environment. This process resulted in the fabrication of two MIM device structures, Al/BST/ITO/glass and Al/BST/TiO_2_/ITO/glass, as schematically illustrated in Figure 1.

To analyze the crystalline structure of the Ba_0.6_Sr_0.4_TiO_3_ (BST) thin films, X-ray diffraction (XRD) measurements were performed over a 2θ scan range of 20° to 60°. The surface morphology and cross-sectional structure of the films were examined using a scanning electron microscope (SEM). The current–voltage (*I–V*) switching behavior of the Al/BST/ITO/glass and Al/BST/TiO_2_/ITO/glass structure RRAM devices was characterized using an Agilent B1500 semiconductor parameter analyzer to evaluate its resistive switching performance. The underlying carrier conduction mechanisms in both the set and reset states were thoroughly investigated and discussed. Finally, the optical transmittance of the fabricated Al/BST/ITO/glass and Al/BST/TiO_2_/ITO/glass structure RRAM devices was measured across the visible spectrum (400–800 nm) using a UV–VIS spectrophotometer, confirming its potential for transparent memory applications.

## 3. Results and Discussion

The crystalline structure of the Ba_0.6_Sr_0.4_TiO_3_ (BST) thin films was characterized using glancing incidence X-ray diffraction (GIXRD) to enhance surface sensitivity and accurately determine crystallographic phases. Measurements were performed on an X-ray diffractometer operated at 45 kV accelerating voltage and 120 mA tube current. A copper anode (Cu Kα radiation, λ = 1.54184 Å) was used as the X-ray source. The scans were conducted over a 2θ range of 20–80° with a fixed incidence angle of 2°. A step size of 0.05° and a dwell time of 3 s per step were employed to ensure high-resolution diffraction signal acquisition.

**Figure 2:**
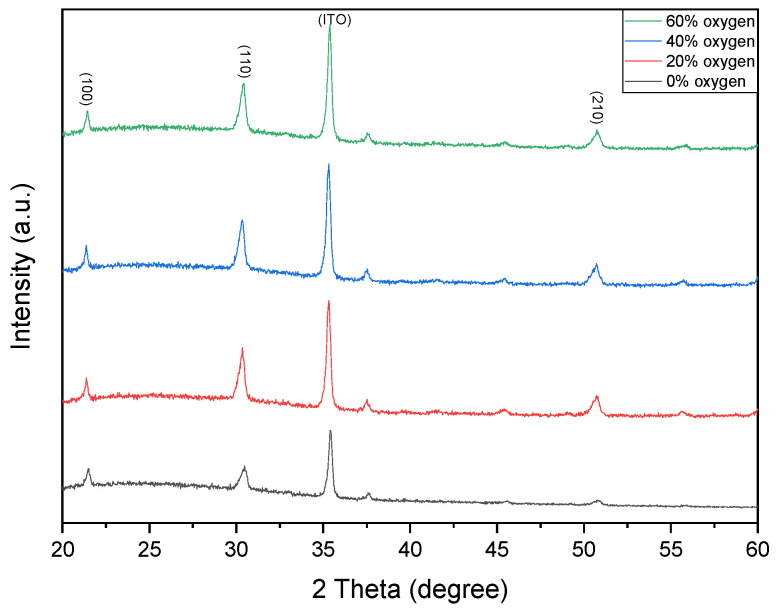
The XRD patterns of as-deposited Ba_0.6_Sr_0.4_TiO_3_ (BST) thin films for different oxygen-to-argon ratio gas composition deposition parameters.

Figure 2 shows the XRD patterns of Ba_0.6_Sr_0.4_TiO_3_ thin films deposited under different oxygen concentrations. The dominant crystalline phases are identified at the (110) and (210) orientations. Notably, the variation in oxygen concentration does not induce a shift in the diffraction peaks, with the main peaks consistently located at approximately 31° and 52° (2θ). Figure 2 present the XRD patterns of Ba_0.6_Sr_0.4_TiO_3_ thin films deposited under oxygen concentrations of 0% and 20% (argon-balanced), respectively. Both samples exhibited polycrystalline structures. The 0% oxygen film displayed randomly oriented diffraction peaks indexed to the (100), (110), and (210) planes. In contrast, the 20% oxygen film exhibited a pronounced (100) preferred orientation, along with a weaker (110) reflection, indicating improved crystallographic texture. Furthermore, the 20% oxygen film demonstrated narrower FWHM values, higher peak intensities, and enhanced crystallinity compared to the 0% oxygen sample. Figure 2 present the XRD patterns of Ba_0.6_Sr_0.4_TiO_3_ thin films deposited under oxygen-to-argon ratios of 0% and 40%, respectively. Both films exhibited polycrystalline structures. The 0% oxygen film showed randomly oriented diffraction peaks, whereas the 40% oxygen film displayed pronounced reflections at (100), (110), and (210), with a strong preferred orientation along the (110) plane. This preferential orientation, confirmed by narrower FWHM values and high peak intensities, indicates enhanced crystallinity resulting from improved oxidation during deposition. Figure 2 show the XRD patterns of BST thin films deposited under O_2_/Ar ratios of 40% and 60%. Both films exhibited polycrystalline structures. The 40% oxygen film displayed diffraction peaks at (100), (110), and (210), with (110) being the preferred orientation. For the 60% oxygen condition, the (110) peak intensity decreased slightly; however, the narrower FWHM confirmed improved crystallinity, which is attributed to the optimized oxygen incorporation into the oxide film.

**Figure 3:**
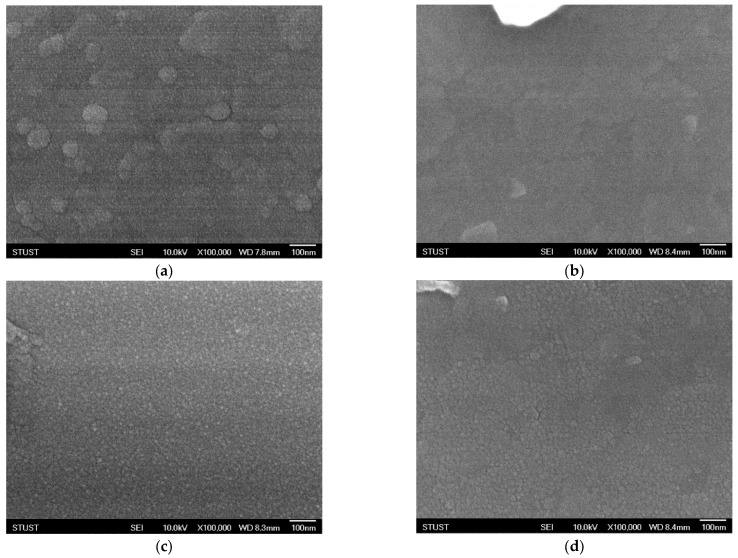
The FE-SEM images of as-deposited Ba_0.6_Sr_0.4_TiO_3_ thin films for different oxygen-to-argon ratio gas composition deposition parameters: (**a**) 0%, (**b**) 25%, (**c**) 40%, and (**d**) 60% conditions.

As shown in Figure 3, the surface morphology of the deposited Ba_0.6_Sr_0.4_TiO_3_ films exhibits significant variations under different oxygen concentrations, as observed through scanning electron microscopy (SEM). Oxygen flow during sputtering plays a crucial role in determining the nucleation process, surface roughness, and grain growth dynamics of oxide-based thin films. Even small changes in oxygen partial pressure can substantially alter the microstructural evolution of the films. At an oxygen concentration of 40%, the SEM images reveal the formation of well-defined grains with clearly distinguishable boundaries. The grains appear to be relatively uniform in size and distribution, indicating that this oxygen level provides a favorable balance between the sputtering rate and the oxidation process. Adequate oxygen incorporation ensures proper stoichiometry of the film and promotes crystallization, leading to enhanced grain visibility and improved crystalline quality. Such microstructural features are typically correlated with higher film density and better electrical properties, which are critical for resistive switching applications. However, when the oxygen concentration is further increased to 60%, a noticeable deterioration in grain uniformity is observed. Excess oxygen flow disrupts the delicate balance between metal ion sputtering and oxide formation, resulting in non-uniform grain growth. The grain sizes become inconsistent, with some regions showing abnormally large grains while others remain relatively fine. This irregularity suggests over-oxidation, which may lead to the formation of oxygen-rich phases, secondary defects, or even amorphous regions within the film. Such conditions can degrade the electrical performance of the device by increasing leakage current paths, introducing localized states, and reducing overall stability. In general, these SEM observations highlight the sensitivity of thin-film growth to oxygen concentration during deposition. An optimal oxygen ratio is essential to control the microstructural evolution, achieve uniform grain morphology, and ultimately ensure reliable device performance. Both under-oxidation and over-oxidation can negatively impact the crystallinity and uniformity of the films, underscoring the importance of process optimization in thin-film fabrication for resistive memory devices.

**Figure 4:**
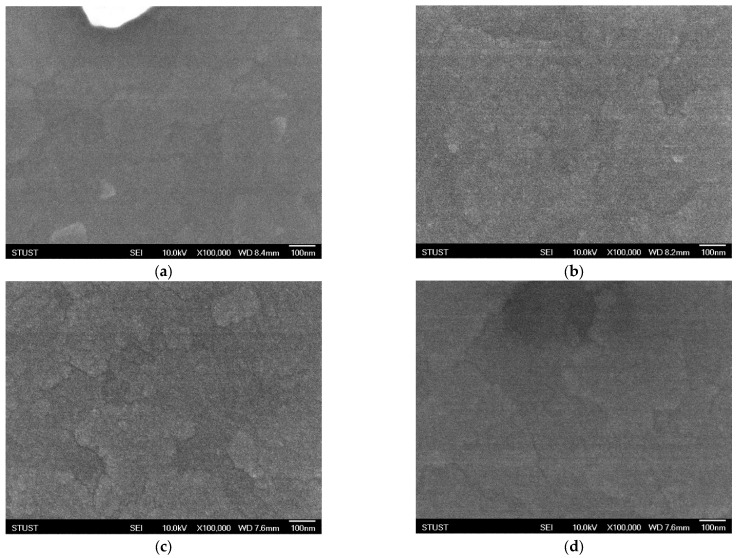
The FE-SEM images of as-deposited Ba_0.6_Sr_0.4_TiO_3_ thin films for 20% oxygen-to-argon ratio gas composition deposition parameters under (**a**) non-treated, (**b**) 450 °C, (**c**) 500 °C, and (**d**) 550 °C RTA post-treatment conditions.

Figure 4 shows the SEM images of thin films deposited under an oxygen concentration of 20% and subjected to different annealing temperatures. After annealing at 450 °C, the SEM results exhibit little difference compared with the as-deposited sample, indicating that no significant morphological changes occurred at this temperature. To further investigate the effect of thermal treatment, the annealing temperature was increased to 500 °C. As shown in Figure 4c, distinct grain evolution can be observed after annealing, with pronounced variations in surface height and morphology. Such uneven crystallinity suggests the presence of defects, which may cause instability in the conductive filament formation process, leading to frequent filament rupture or unreliable switching behavior. In an attempt to suppress these defects, the annealing temperature was further raised to 550 °C. However, as shown in Figure 4d, partial surface melting was observed, indicating severe degradation of the thin-film morphology. From an electrical standpoint, the device performance at 550 °C is expected to be even worse than that at 500 °C. These findings clearly demonstrate that under insufficient oxygen concentration during deposition, the thin films exhibit inherent instability, which cannot be effectively compensated by post-deposition annealing.

**Figure 5:**
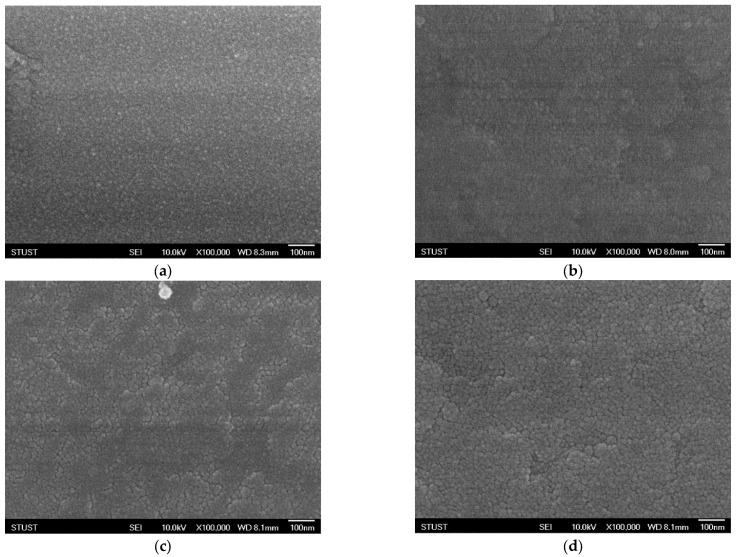
The FE-SEM images of as-deposited Ba_0.6_Sr_0.4_TiO_3_ thin films for 40% oxygen-to-argon ratio gas composition deposition parameters under (**a**) non-treated, (**b**) 450 °C, (**c**) 500 °C, and (**d**) 550 °C RTA post-treatment conditions.

Figure 5 presents the SEM images of thin films deposited under an oxygen concentration of 40% and subjected to different annealing temperatures. After annealing at 450 °C, the surface morphology of the crystalline structure exhibits slight changes; however, no significant modification is observed compared with the as-deposited sample. When the annealing temperature is increased to 500 °C, as shown in Figure 5c, the grains undergo a more noticeable transformation, indicating enhanced crystallization and structural reorganization. To further identify the optimal annealing temperature, the treatment was continued at a higher temperature of 550 °C. As illustrated in Figure 5d, the grains become denser and more compact, demonstrating that an appropriate annealing temperature not only repairs structural defects but also promotes effective recrystallization, thereby improving the overall quality of the thin film.

Figure 5 shows the SEM images of thin films deposited under an oxygen concentration of 60% and subjected to different annealing treatments. In the as-deposited state, the film exhibits noticeable crystalline agglomerations with a block-like morphology. Compared with the case at 40% oxygen concentration, this blocky crystallization is more pronounced, which may be attributed to the excessive oxygen flow during deposition. After annealing at 450 °C, the grain size becomes significantly smaller; however, the crystalline features are much less distinct. This diminished crystallinity suggests a higher likelihood of instability in conductive filament formation, which may adversely affect the electrical performance of the device. To further investigate the influence of thermal treatment, the annealing temperature was increased to 500 °C. As shown in Figure 5c, granular features re-emerge on the thin-film surface, but their distribution is highly non-uniform, indicating inhomogeneous recrystallization and potential structural defects. To evaluate whether higher annealing could improve the morphology, the annealing temperature was further raised to 550 °C. As presented in Figure 5d, grain growth is observed at this stage; however, partial surface melting also appears, which severely compromises the integrity of the film. Based on these results, it can be inferred that 500 °C is the most suitable annealing temperature under 60% oxygen concentration, balancing grain formation with minimal surface degradation.

**Figure 6:**
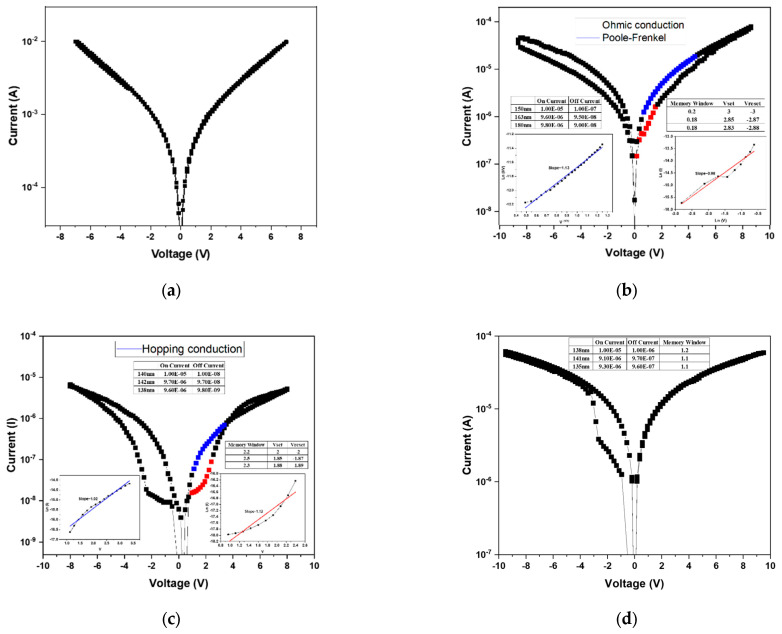
The *I–V* curves of as-deposited Ba_0.6_Sr_0.4_TiO_3_ thin films for different oxygen-to-argon ratio gas composition deposition parameters: (**a**) 0%, (**b**) 25%, (**c**) 40%, and (**d**) 60% conditions.

After the forming process, the BST thin films RRAM device successfully established the conductive filaments, enabling a low-resistance state (LRS). Subsequently, a negative bias was applied to induce the reset process, during which the conductive filament was ruptured, transitioning the device from a LRS to a high-resistance state (HRS). By repeatedly applying forward and reverse voltage sweeps, the memory window of the RRAM device was characterized. The *I–V* switching characteristics under bipolar switching are shown in Figure 6. In the absence of oxygen during deposition (Figure 6a), no memory window was observed, as excessive oxygen vacancies prevented stable filament formation and hindered differentiation between the HRS and LRS. When the oxygen concentration was increased to 20% (Figure 6b), a discernible memory window appeared, suggesting partial compensation of oxygen vacancies. Although the on/off ratio was below one order of magnitude, the results confirmed that higher oxygen concentrations could further enhance switching performance. At an oxygen concentration of 40% (Figure 6c), the memory window was significantly improved, with an on/off ratio of approximately 2 and an operating voltage of around 2 V. This indicates that appropriate oxygen incorporation optimizes the defect balance and filament dynamics, leading to enhanced device performance. To further examine this trend, deposition was carried out at an oxygen concentration of 60% (Figure 6d). However, the switching behavior transformed from bipolar to unipolar. This transition was attributed to the excessive oxygen supply, which overcompensated vacancies and prevented effective rupture of conductive filaments, thereby suppressing the HRS recovery. From these results, it was concluded that 40% oxygen concentration represents the optimal deposition condition, yielding stable bipolar resistive switching with a clear memory window and reliable operation.

Since the sample deposited without oxygen (0% O_2_) exhibited no observable memory window, its conduction behavior was not analyzed in the discussion. Figure 6b presents the conduction mechanism fitting results for the device fabricated under 20% oxygen concentration. Based on Equation (1), the relationship between ln(I) and ln(V) was plotted to identify ohmic conduction, where a slope close to 1 confirms ohmic behavior. The analysis indicates that, in the HRS region at low applied voltage, the conduction follows the ohmic conduction mechanism.

The electrical current density of the ohmic conduction mechanism was calculated using Equation (1):

(1) $$J=E_{i}exp\left(\frac{-△E_{ac}}{kT}\right)$$

$E_{i}$ is Electric field in the insulator, $△E_{ac}$ is Electron activation energy, k is Boltzmann constant, and T is Temperature [15].

The electrical current density of the Poole–Frenkel conduction mechanism was calculated using Equation (2):

(2) $$J=\frac{9\varepsilon_{i}uV^{2}}{8d^{3}}=\left(\frac{9\varepsilon_{i}u}{8d^{3}}\right)E_{i}^{2}$$

$\varepsilon_{i}$ is Dielectric constant, u is Carrier mobility, d is Insulator thickness, and $E_{i},$ *V* is Electric field and voltage across the insulator [25,26,27,28].

Subsequently, by rearranging Equation (2), a plot of √V versus ln(I/V) was constructed. A linear relationship in this plot corresponds to the Poole–Frenkel emission mechanism. The LRS fitting results revealed that conduction is dominated by the Poole–Frenkel emission, implying that electrons were trapped and released through defect states within the oxide film.

For the 40% oxygen concentration sample, the conduction mechanism analysis is shown in Figure 6c. After rearranging Equation (3), V versus ln(I) was plotted, where a linear slope indicates hopping conduction. Both the HRS and LRS exhibited hopping behavior, suggesting that numerous localized defect states exist within the film, allowing charge carriers to hop between adjacent traps.

(3) $$J=\alpha \;nvq\;\exp\left(\frac{-U}{kT}+\frac{q\alpha E}{kT}\right)$$

*J* is Current density, α is Average jump distance, *n* is Electron density of the conduction band in the film, *v* is Frequency of electron vibrations due to heat in the defect, *U* is Energy difference from the defect to the conduction band, and *E* is Equivalence strength [25,26,27,28].

The 60% oxygen concentration sample exhibited a transition from bipolar to unipolar switching, and therefore only the unipolar conduction behavior was analyzed. As shown in Figure 6d, using Equation (1), the ln(V)–ln(I) plot revealed linear slopes in both HRS and LRS regions, confirming ohmic conduction. This indicates that electron transport occurs primarily through direct conduction across the oxide layer rather than via trap-assisted mechanisms.

**Figure 7:**
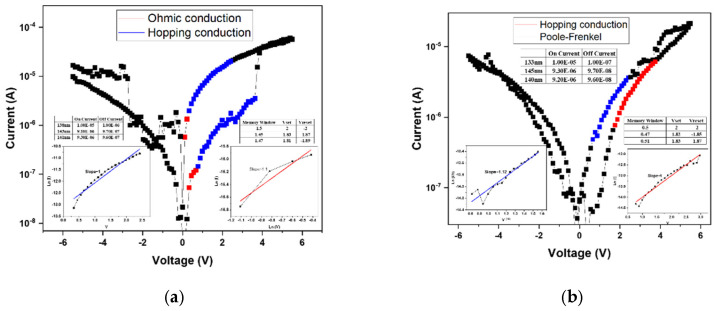
The *I–V* curves of as-deposited Ba_0.6_Sr_0.4_TiO_3_ thin films for 20% oxygen-to-argon ratio gas composition deposition parameters under (**a**) 500 °C and (**b**) 550 °C RTA post-treatment conditions.

Figure 7a presents the linear fitting results of the device annealed at 500 °C. At low applied voltages, the conduction behavior follows the ohmic conduction mechanism, which occurs when a portion of the injected electrons gain sufficient energy to overcome the potential barrier and move freely through the insulating layer. As the applied voltage increases, the conduction mechanism transitions to hopping conduction, attributed to the presence of numerous shallow defect states within the film. These localized states facilitate carrier transport through successive hopping events. According to the fitting results, both the high-resistance state (HRS) and low-resistance state (LRS) are dominated by hopping conduction, governing the resistive switching between “0” and “1” states. Figure 7 shows the linear fitting results for the device annealed at 550 °C, exhibiting a similar trend to that observed at 500 °C. In this case, the HRS conduction is dominated by hopping transport, while the LRS conduction follows the Poole–Frenkel emission mechanism. The latter indicates that defect states within the insulating layer capture and release electrons during carrier transport, resulting in current formation through trap-assisted transitions. Moreover, the electrical characteristics reveal irregular filament formation and an incomplete memory window, likely caused by insufficient oxygen concentration during film growth. The resulting excess oxygen vacancies hinder the formation of stable conductive filaments, thereby degrading resistive switching performance.

**Figure 8:**
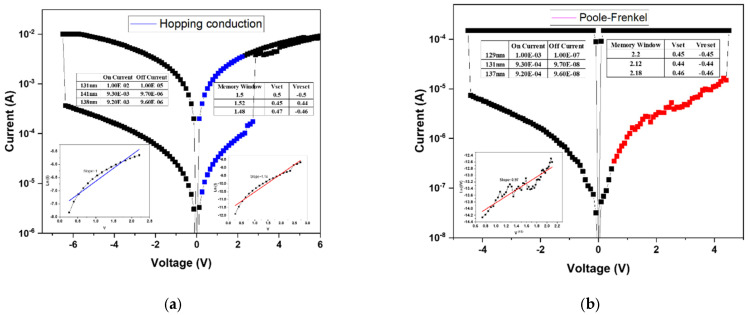
The *I–V* curves of as-deposited Ba_0.6_Sr_0.4_TiO_3_ thin films for 40% oxygen-to-argon ratio gas composition deposition parameters under (**a**) 500 °C and (**b**) 550 °C RTA post-treatment conditions.

Figure 8a presents the linear fitting results of the device annealed at 500 °C, where both the conductive filament formation and the memory window are well-defined. This indicates that an appropriate combination of oxygen concentration and annealing temperature effectively stabilizes the *I–V* characteristics of the RRAM device. According to the fitting analysis, both the high-resistance state (HRS) and low-resistance state (LRS) follow the hopping conduction mechanism, suggesting that carrier transport occurs through electron hopping among shallow defect states within the oxide film. Figure 8b shows the linear fitting results for the device annealed at 550 °C. In this case, the HRS conduction mechanism transitions to the Poole–Frenkel emission, whereas at 500 °C it was dominated by hopping conduction. This transition implies that the higher annealing temperature promotes grain regrowth and defect healing within the oxide layer. As a result, trap density is reduced, altering the dominant conduction mechanism from defect-mediated hopping to trap-assisted Poole–Frenkel conduction. This observation highlights the strong correlation between microstructural evolution and carrier transport behavior in annealed oxide-based RRAM devices.

**Figure 9:**
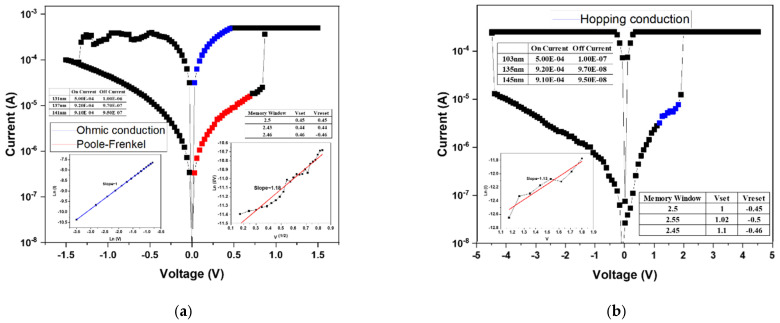
The *I–V* curves of as-deposited Ba_0.6_Sr_0.4_TiO_3_ thin films for 60% oxygen-to-argon ratio gas composition deposition parameters under (**a**) 500 °C and (**b**) 550 °C RTA post-treatment conditions.

Figure 9a presents the linear fitting analysis of the device annealed at 500 °C. Compared with the sample annealed at 450 °C, the memory window shows a notable improvement. Based on the fitting results, the high-resistance state (HRS) follows the Poole-Frenkel conduction mechanism, while the low-resistance state (LRS) is governed by ohmic conduction. This transition suggests that at higher bias, trap-assisted electron emission dominates in the insulating layer, whereas in LRS, direct conduction occurs through the established conductive filaments. The corresponding *I–V* characteristics indicate a memory window of approximately two orders of magnitude, confirming enhanced switching uniformity and stability after annealing. Figure 9b shows the fitting results for the device annealed at 550 °C. In this case, the HRS is dominated by hopping conduction, differing from the Poole–Frenkel mechanism observed in the 40% oxygen concentration sample annealed at the same temperature. This variation is attributed to the excess oxygen content introduced during film deposition, which, while improving surface smoothness, also leads to the formation of shallow defect states within the oxide matrix. These localized defect sites facilitate electron transport via hopping between adjacent traps, thereby resulting in a hopping-dominated conduction mechanism.

**Figure 10:**
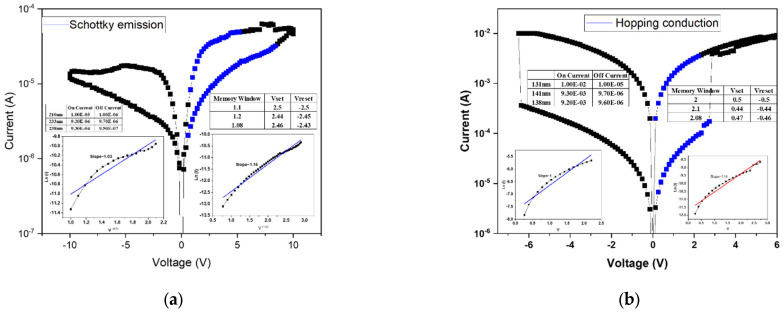
The *I–V* curves of as-deposited 40%–Ba_0.6_Sr_0.4_TiO_3_ thin films under 500 °C RTA post-treatment condition for devices (**a**) with and (**b**) without an electron transport layer.

The electrical characteristics of the devices are shown in Figure 10, where the results are compared with those obtained under 40% oxygen concentration and an annealing temperature of 500 °C. A distinct difference was observed between the two cases. Without the electron transport layer (ETL), the device exhibits a unipolar resistive switching behavior. However, after introducing the ETL, the switching mode transitions from unipolar to bipolar operation. Moreover, the leakage current is significantly reduced—from approximately 10^−3^ A to 10^−5^ A, following the incorporation of the ETL. This improvement indicates that the addition of the electron transport layer effectively suppresses leakage pathways, likely by acting as an energy barrier or defect passivation layer at the interface, thereby enhancing the overall switching stability and energy efficiency of the RRAM device.

The electrical current density of the Schottky conduction mechanism was calculated using Equation (4):

(4) $$J_{SE}=A^{*}T^{2}exp\left(\frac{\beta_{SE}\sqrt{E}}{kT}\right)$$

$C_{RD}$ is Effective Richardson constant, q is Elementary charge of the carrier, $m^{*}$ is Effective mass of the carrier, k is Boltzmann constant, h is Insulator thickness, $\Phi_{B}$ is Barrier height, T is Temperature, $\varepsilon_{0}$ is Vacuum permittivity, $\varepsilon_{r}$ is Dynamic dielectric constant (at high frequency), and E is Electric field in the insulator [15].

The conduction mechanism analysis of as-deposited 40%-Ba_0.6_Sr_0.4_TiO_3_ thin films under 500 °C RTA post-treatment condition with regard to using an electron transport layer is also presented in Figure 10. It can be observed that upon the introduction of the electron transport layer (ETL), both the high-resistance state (HRS) and low-resistance state (LRS) exhibit Schottky emission behavior. This indicates that the inclusion of an additional insulating layer increases the potential barrier height, thereby requiring charge carriers to overcome this barrier during transport to generate measurable current flow. Consequently, the resistance switching process, corresponding to data storage between logic states “0” and “1,” is dominated by carrier injection across the Schottky barrier. To provide a clearer understanding of this process, the corresponding energy band diagram illustrating electron transport across the MIM structure is shown in Figure 10.

**Figure 11:**
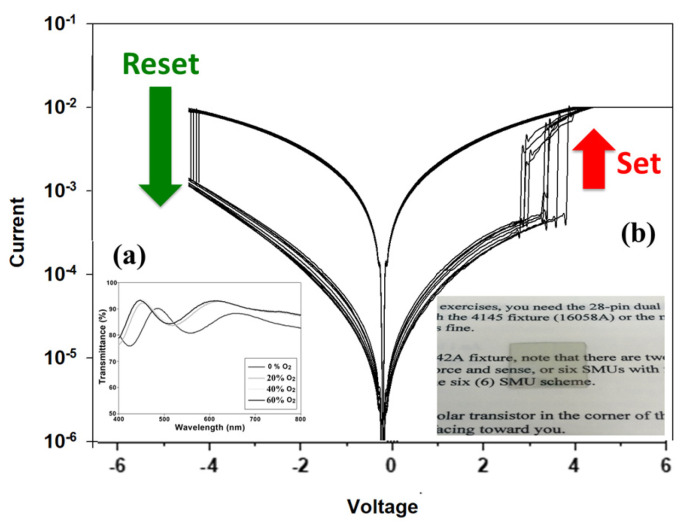
The 100 times *I–V* curves of 500 °C-treated Ba_0.6_Sr_0.4_TiO_3_ thin films for (**a**) UV–VIS properties and (**b**) transmittance images.

Figure 11 presents the 100 times *I–V* curves of 500 °C-treated Ba_0.6_Sr_0.4_TiO_3_ thin film RRAM devices for the LRS/HRS in a set/reset state. For the visible wavelength range from 400 to 800 nm, the transmittance properties of 500 °C-treated Ba_0.6_Sr_0.4_TiO_3_ thin films RRAM devices were measured, as shown in Figure 11a. For the 0% oxygen growth procedure parameters, the transmittance efficiency was about 88% for all visible wavelength ranges. An optimal transmittance efficiency of 90% for different oxygen growth procedure parameters was observed. Finally, the excellent transparency efficiency of the 500 °C-treated Ba_0.6_Sr_0.4_TiO_3_ thin films RRAM, as observed by optical images, is shown in Figure 11b.

**Figure 12:**
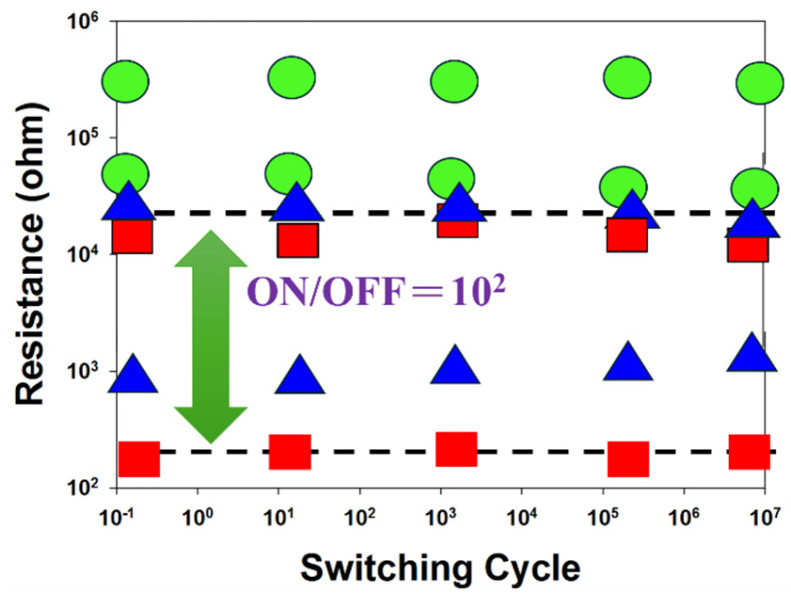
The resistance value versus switching cycle curves of the Ba_0.6_Sr_0.4_TiO_3_ thin films RRAM devices (red: 20% oxygen content, blue: 40% oxygen content, green: 60% oxygen content).

**Figure 13:**
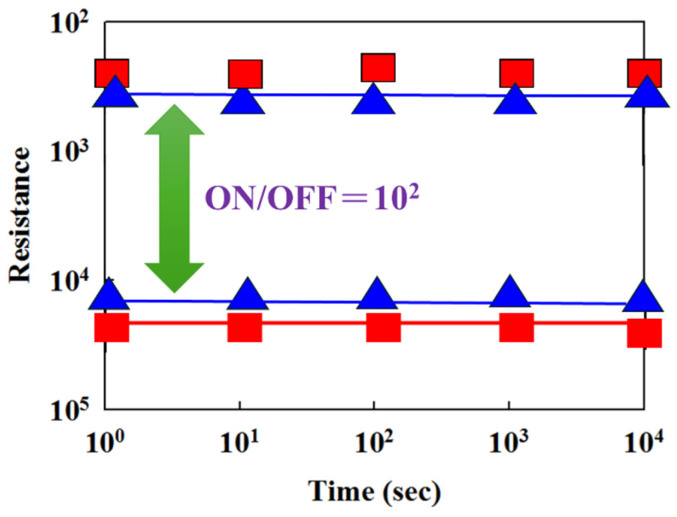
The resistance value versus time curves of 40%-Ba_0.6_Sr_0.4_TiO_3_ thin films RRAM devices (red: 500 °C, blue: 550 °C RTA-treated).

Figure 12 presents the resistance versus switching cycle characteristics of BST RRAM devices for different deposition parameters, where red, blue, and green curves represent 20% oxygen content, 40% oxygen content, and 60% oxygen content, respectively. The extrapolated data indicate stable on/off switching behavior over a period exceeding 10^2^ s. Figure 13 illustrates the resistance retention and endurance performance of the BST RRAM devices, evaluated through repeated switching cycles. The retention characteristics for different deposited parameters for RTA-treated samples were assessed to determine their reliability for non-volatile memory applications. According to the extrapolated results, both devices maintained stable on/off resistance ratios without significant degradation for durations exceeding 10^2^ s.

**Figure 14:**
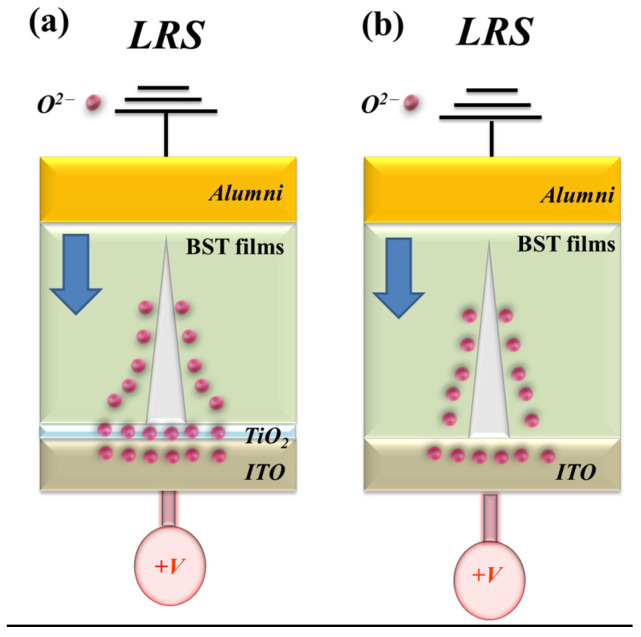
The initial metallic filament path model of the Ba_0.6_Sr_0.4_TiO_3_ thin films RRAM devices under a 500 °C RTA post-treatment condition for devices (**a**) with and (**b**) without an electron transport layer.

Figure 14 illustrates the formation and evolution of oxygen vacancies at the interface between the ITO/TiO_2_ electron transport layer and the Ba_0.6_Sr_0.4_TiO_3_ (BST) thin film within the RRAM device. During the low-resistance state (LRS), oxygen vacancies progressively accumulate along the conductive filament path. Under a high positive bias applied to the bottom electrode, a localized oxidation–reduction reaction occurs along the filament, dynamically modulating the resistive switching behavior. The redistribution of oxygen ions near the electrode interface critically influences filament growth, rupture, and stability. The schematic in Figure 14 depicts the stepwise formation and conduction pathway of metallic filaments during the SET process, as well as the corresponding charge–transport mechanisms in BST-based RRAM devices incorporating ITO/TiO_2_ layers. For these BST-based structures, the presence of the electron transport layer (ETL) leads predominantly to Schottky-type conduction under low bias conditions. This behavior is attributed to interfacial defect states and the built-in potential barrier at the TiO_2_/BST interface, which effectively suppress leakage current and enhance the switching uniformity of the device [29,30].

**Figure 15:**
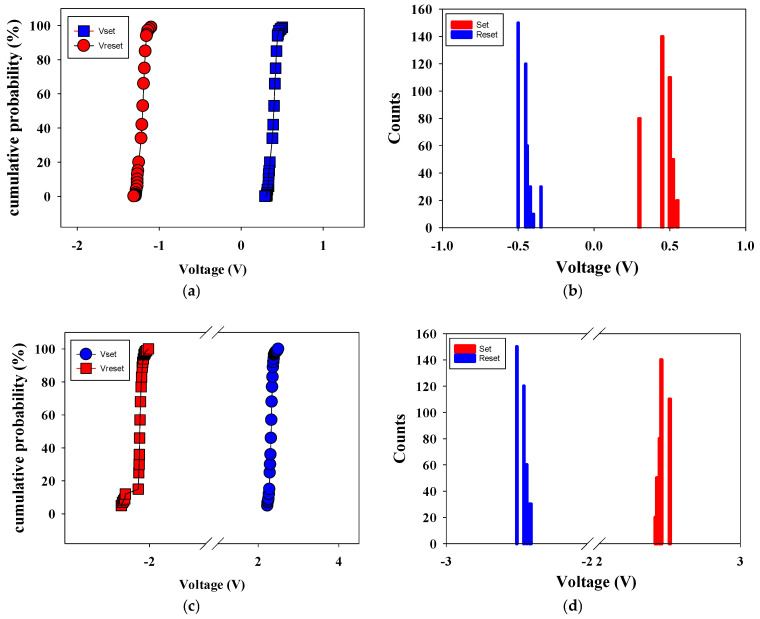
The set/reset voltage statistics versus counts distribution, and cumulative probability of the 500 °C-treated Ba_0.6_Sr_0.4_TiO_3_ thin films RRAM devices (**a**) with an ETL, (**b**) with an ETL, (**c**) without an ETL, and (**d**) without an ETL.

Figure 15 shows the set/reset voltage statistics versus counts distribution, and cumulative probability of 500 °C-treated Ba_0.6_Sr_0.4_TiO_3_ thin films RRAM devices. Figure 15a,b presents the statistical results of distribution of the set/reset voltage measured in RRAM devices without an electrical transfer layer. In Figure 15a, the statistical results of cumulative probability for the resistive switching properties of the RRAM devices were also observed. In addition, the set and reset voltage values were found for the range of applied voltage of −1 to 0.5 V in the counts distribution results shown in Figure 15b. Figure 15c,d present the statistical results of distribution of the set/reset voltage measured in RRAM devices with an electrical transfer layer. In Figure 15c, the statistical results of cumulative probability for the resistive switching properties of the RRAM devices were also observed. In addition, the set and reset voltage values were found for the range of applied voltage of −2 to 2 V in the counts distribution results shown in Figure 15d. As shown by the set statistical results in Figure 15, using the electrical transfer layer correlates with an increase in the set voltage. This suggests that high set/reset voltage values can be attributed to the thickness of Ba_0.6_Sr_0.4_TiO_3_ thin film in the set/reset process.

**Figure 16:**
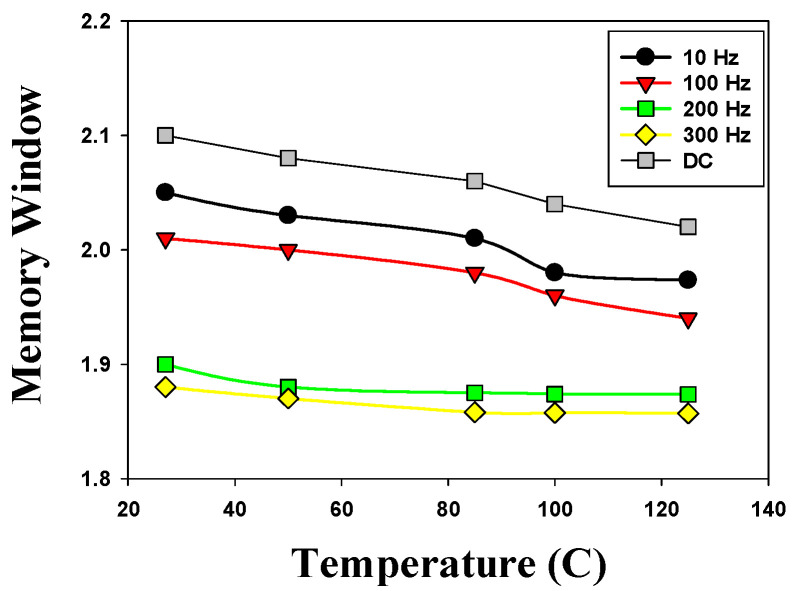
The memory window versus temperature curves of the 500 °C-treated Ba_0.6_Sr_0.4_TiO_3_ thin films RRAM device for the different AC and DC operations.

Figure 16 presents the memory window versus temperature curves of the 500 °C-treated Ba_0.6_Sr_0.4_TiO_3_ thin films RRAM device for the different AC and DC operations. In 27 °C temperature, the 500 °C-treated Ba_0.6_Sr_0.4_TiO_3_ thin films RRAM device exhibited a 2.1 ratio of memory window in DC operation. Regarding the effect of temperature dependency on device performance, the memory window of the 500 °C-treated Ba_0.6_Sr_0.4_TiO_3_ thin films RRAM device was slightly decreased by the AC cycle increasing from 10 Hz to 300 Hz. The experimental results showed that the device performance characteristics of thin films’ memory components maintain stability. In addition, we also found that the memory window decreased slightly under different temperature measurements.

Table 1 presents the Vset and Vreset values for a comparison of the structure and the transmittance ratio of the various ITO films RRAM devices. In this study, the Vset value, Vreset value, and memory window in the case of the electrical conduction mechanism were extracted from the *I–V* curves and compared with the values observed in the literature, as summarized in Table 1. The Vset/Vrest values of our thin films RRAM devices were about 1.5 V and −1.5 V, respectively. As seen in Table 1, the 500 °C-treated Ba_0.6_Sr_0.4_TiO_3_ thin films exhibited high transmittance properties of 95% and lower Vset/Vrest values than others. When comparing area dependency as a function of device performance, we observe that the memory window variations of the RRAM device were affected by size variations within the 10 to 15 nm scale in DC/AC cycles [31]. Above the 15 nm scale of the RRAM devices’ size, the set/rest voltage and memory window maintained stable performance characteristics. In addition, the experimental results show a drastic change in memory performance characteristics on a scale of less than 10–15 nm [31,32].

## 4. Conclusions

Ba_0.6_Sr_0.4_TiO_3_ (BST) thin films were deposited on ITO/glass substrates using the radio-frequency magnetron sputtering, with the oxygen partial pressure systematically varied to optimize film growth. Structural, morphological, and electrical analyses were performed to examine the conduction behavior. Aluminum-top electrodes were deposited through thermal evaporation to form metal–insulator–metal (MIM) resistive random-access memory (RRAM) structures. Post-deposition rapid thermal annealing (RTA) at various temperatures enhanced crystallinity and reduced defect density, with 500 °C identified as the optimal condition based on electrical performance.

BST films deposited under 40% oxygen concentration exhibited stable resistive switching, with an on/off ratio of 1. Increasing oxygen concentration induced a transition from bipolar to unipolar switching. The 500 °C RTA-treated films showed superior characteristics, with set/reset voltages of 2 V and −2 V, respectively, and a memory window of two orders of magnitude. Fitting analysis confirmed hopping conduction as the dominant mechanism.

To improve switching uniformity, an electron transport layer (ETL) was incorporated beneath the BST layer, forming a metal–insulator–insulator–metal (MIIM) structure. This configuration exhibited asymmetric switching (Vset and Vreset of 6 V), governed by Schottky emission in both resistance states. Compared with the conventional MIM device, the ETL-enhanced structure demonstrated improved filament stability and lower leakage current. These results highlight the potential of BST-based RRAM for reliable, CMOS-compatible, next-generation, non-volatile memory applications.

## Figures and Tables

**Table 1:** The Vset and Vreset values for comparing the structure and the transmittance ratio of the various ITO films RRAM devices.

Row	Structure	Vset	Vreset	Memory Window	Transmittance %	Ref.
1	ITO/Nd_2_O_3_/ITO	1	−1	2	90	[30]
2	ITO/HfO_2_/ITO	6	−4	2	80	[33]
3	ITO/ZnO/ITO	1	−1	2	80	[34]
4	ITO/Al_2_O_3_/ITO	1.5	−1.5	2.5	85	[35]
6	ITO/CeO_2_/ITO	2	−2	1	78	[36]
5	ITO/BST/ITO	1.5	−1.5	2.5	95	This Work

## Data Availability

The original contributions presented in this study are included in the article. Further inquiries can be directed to the corresponding authors.
